# Immunoproteomic Analysis of Proteins Expressed by Two Related Pathogens, *Burkholderia multivorans* and *Burkholderia cenocepacia*, during Human Infection

**DOI:** 10.1371/journal.pone.0080796

**Published:** 2013-11-15

**Authors:** Minu Shinoy, Ruth Dennehy, Lorraine Coleman, Stephen Carberry, Kirsten Schaffer, Máire Callaghan, Sean Doyle, Siobhán McClean

**Affiliations:** 1 Centre of Microbial Host Interactions, ITT Dublin, Tallaght, Dublin, Ireland; 2 Centre of Applied Science for Health, ITT Dublin, Tallaght, Dublin, Ireland; 3 Department of Biology, National University of Ireland, Maynooth, Co Kildare, Ireland; 4 Department of Microbiology, St. Vincent's University Hospital, Elm Park, Dublin, Ireland; Queens University Belfast, Ireland

## Abstract

*Burkholderia cepacia* complex (Bcc) is an opportunistic bacterial pathogen that causes chronic infections in people with cystic fibrosis (CF). It is a highly antibiotic resistant organism and Bcc infections are rarely cleared from patients, once they are colonized. The two most clinically relevant species within Bcc are *Burkholderia cenocepacia* and *Burkholderia multivorans*. The virulence of these pathogens has not been fully elucidated and the virulence proteins expressed during human infection have not been identified to date. Furthermore, given its antibiotic resistance, prevention of infection with a prophylactic vaccine may represent a better alternative than eradication of an existing infection. We have compared the immunoproteome of two strains each from these two species of Bcc, with the aim of identifying immunogenic proteins which are common to both species. Fourteen immunoreactive proteins were exclusive to both *B. cenocepacia* strains, while 15 were exclusive to *B. multivorans*. A total of 15 proteins were immunogenic across both species. DNA-directed RNA polymerase, GroEL, 38kDa porin and elongation factor-Tu were immunoreactive proteins expressed by all four strains examined. Many proteins which were immunoreactive in both species, warrant further investigations in order to aid in the elucidation of the mechanisms of pathogenesis of this difficult organism. In addition, identification of some of these could also allow the development of protective vaccines which may prevent colonisation.

## Introduction

*Burkholderia cepacia* complex (Bcc) is a heterogenous group of Gram negative bacteria comprising at least 17 species. It is one of a number of opportunistic pathogens that causes chronic respiratory infections in people with the genetically inherited diseases cystic fibrosis (CF) and chronic granulomatous disease (CGD). In contrast to other pathogens associated with CF, such as *Pseudomonas aeruginosa*, a subgroup of Bcc colonised patients can develop a fatal necrotizing pneumonia associated with septicaemia referred to as “cepacia syndrome” [1]. Bcc is particularly difficult to treat due to its inherent antimicrobial resistance and once acquired it is rarely eradicated. There are two species that are of particular interest, *Burkholderia cenocepacia* which is the most virulent species and *Burkholderia multivorans*, which is currently the most frequently isolated Bcc species from newly colonised CF patients [2,3]. Together these species account for 85% to 95% of Bcc CF infections. Bcc are typically found in water and soil and can survive for prolonged periods in moist environments [4]. Although Bcc has been associated with patient-to-patient transmission, these have now been largely minimised by strict segregation measures [5]. The majority of new acquisitions of *B. multivorans* are considered to be from the environment [4]. We and others have shown that environmental Bcc strains have the potential to be as virulent as clinical strains [6,7]. 

 The mechanisms of colonisation and pathogenesis of Bcc are still poorly understood [8] and many virulence factors remain unidentified. In particular, the virulence factors expressed by this pathogen during human infection have not been elucidated to date. Well known virulence factors are generally strongly immunogenic (for example, *Streptococcus pneumonia* antigens [9]), therefore a key objective of this study to identify novel Bcc virulence factors that might contribute to the pathogenesis of this organism, using an immunoproteomics approach with patient serum. Membrane proteins were of particular interest as these are exposed to, and make direct contact with, host cells and are therefore among the primary antigen targets of the host immune system. In addition, another aim of this study was to identify immunogenic proteins from both *B. multivorans* and *B. cenocepacia* strains, which would allow the development of protective vaccine antigens to prevent colonisation by both the most commonly acquired species and the most virulent species. Recent developments in proteomics have allowed the analysis and identification of the immunogenic proteins from pathogenic microbes, which has led to the identification of novel antigens in many pathogenic organisms such as *Burkholderia pseudomallei, Pseudomonas aeruginosa, Streptococcus pneumonia, Staphylococcus epidermidis*, and *Neisseria meningitides* [10-14] some of which show promise as potential vaccine targets [15]. Given the high levels of multidrug resistance within Bcc, prevention of colonisation via vaccination may be a more promising approach than eradication of a chronic infection. 

We have examined membrane protein preparations from four different Bcc strains from the International Burkholderia cepacia Working Group panel [16] in order to focus on proteins that are conserved across both species. These four strains represent a piliated strain and non-piliated strain from *B. cenocepacia*, BC-7 and C1394, respectively, and two strains from *B. multivorans*, LMG 13010 and C1962. 

## Materials and Methods

### Bacterial strains and culture conditions

The four bacterial strains used in this study, two *B. multivorans* strains, LMG13010 and C1962, and two *B. cenocepacia* strains, BC-7 (rec IIIA lineage) and C1394 (rec IIIB lineage) and were obtained from the BCCM/LMG, University of Ghent, Belgium and routinely plated on *B. cepacia* specific agar (BCSA) [17]. The bacterial strains were routinely cultured in Luria Bertani (LB) broth at 37°C with orbital shaking (150 rpm). The stationary phase cultures were prepared in 10 L fermenter for the isolation of membrane proteins at 37°C without pH control or anti-foam control with an air supply of 6 L/min. 

### Membrane protein preparation

Membrane proteins from *B. multivorans* strain LMG13010 and C1962 or *B. cenocepacia* strain BC-7 and C1394 were prepared by centrifugation of cells at 5,000 g for 10 min at 4 °C, and resuspended in ice cold PBS containing 5 % CHAPS 3-[(3-cholamidopropyl) dimethyl ammonio]-1-propane sulfonate) and protease inhibitor cocktail. Cell debris was removed 5,000 g for 10 min at 4° C, the supernatants ultracentrifuged at 30,000 g at 4 °C for 30 min and the pellets were resuspended in 10 mL of 2 mM MgCl_2_, 50 mM Tris (pH 8) containing protease inhibitor cocktail and centrifuged under the same conditions. The new pellets were resuspended in 2 % Triton X-100, 50 mM Tris (pH 8) with protease inhibitor cocktail and incubated for 30 min at 40 °C with gentle shaking. The ultracentrifugation was repeated for another hour, the pellets were washed with 50 mM Tris (pH8) buffer with protease inhibitors and ultracentrifuged in the same conditions. The final pellets were resuspended in 50 mM Tris, pH 8 and analysed for protein content using Bradford assay. Membrane preparations were prepared on three independent occasions for each strain. 

### Protein Separation by 2-D gel Electrophoresis

The membrane protein preparations were solubilised for isoelectric focusing (IEF) in rehydration solution (8 M urea, 2 M thiourea, 4 % CHAPS, 1 % Triton, 10 mM Tris base, 65 mM dithiothreitol (DTT) and 0.8 % (v/v) IPG buffer (pH 3-11) and a trace of bromophenol blue. The Immobilised pH gradient strips non-linear (IPG) strips (pH 3-11NL) of 7 cm were rehydrated overnight with 120 μL of the rehydration solution containing 120 µg of proteins. The IEF step was carried out for 3 h focusing with a total voltage of 7,000V applied and the IPG strips equilibrated in reducing buffer for 20 min under agitation in 30 % glycerol, 2 % sodium dodecyl sulphate (SDS), 6 M urea, 50 mM Tris and 2 % DTT. The IPG strips were then alkylated in the same buffer containing 2.5 % iodoacetamide. The IPG strips were placed on 12 % SDS-PAGE gels and the separation was carried out at 110 V. For each individual experiment, three gels were prepared, one of which was stained with Coomassie blue (PageBlue, Fermentas) and the other two were transferred to PVDF membrane for subsequent immunoprobing. Blots were repeated on separate independent membrane preparations for each individual strain. 

### Human sera samples

Sera from seven adult CF patients that had a history of Bcc infection (Bcc+) were pooled to maximise the identification of immunogenic proteins and avoid patient-specific effects. In addition, serum from six adult CF patients that had no history of Bcc infection and that were confirmed as negative for Bcc infection was used as negative controls (Bcc-). These six Bcc negative sera were positive for *P. aeruginosa* antibodies. All subjects gave written informed consent to the use of their serum. Ethics approval was obtained for working with sera from CF patients from the St Vincent’s University Hospital Research Ethics Committee and from the Institute of Technology Tallaght Dublin Research and Ethics Committee. 

### Immunoblotting of bacterial membrane proteins

Prior to transfer of the proteins from gels to the PVDF membrane, the gels were equilibrated in the transfer buffer (25 mM Tris, 192 mM glycine, 20 % (v/v) methanol) for 15 min and protein transfer was performed in a semi-dry Transphor unit (Bio-Rad) at 330 mA for 50 min. The PVDF membrane was then blocked with 5 % BSA and 2 % Marvel^®^ dried milk powder in PBS overnight at 4° C. Pairs of blots were either probed with pooled Bcc+ serum or Bcc- serum, (1;8,000), for 1 h and washed with PBST (0.05 % Tween-20) three times. The blots were incubated with HRP conjugated secondary Ab (1:16,000, Roche) and washed again five times with PBS containing 0.4 % Tween-20. Chemiluminescent detection was carried with Ampli-Cruz detection kit (Santa Cruz Biotechnology, Germany) and exposed to Kodak photographic film. 

### In gel trypsin digestion and MALDI-ToF MS/MS analysis

Protein spots excised from 2-D gels were destained with an equal volume of 100 mM ammonium bicarbonate and acetonitrile. Gel digestion was performed with 13 ng μL^-1^ modified porcine trypsin for 2 h on ice followed by overnight incubation of samples at 37 °C. Tubes were chilled to RT, and gel pieces pelleted using a micro-centrifuge. Aliquots of 2 to 2.5 μL of the supernatant were withdrawn directly from the digest for MALDI-ToF MS/MS analysis with an Ultraflex MALDI-ToF mass spectrometer (BrukerDaltonics), without further extraction. An equal volume of peptide extracts and matrix solution (2 mg mL^-1^ α-cyano-4-hydroxycinnamic acid (CHCA) in 70 % of 0.1 % TFA and 30 % of acetonitrile) were applied on to the target plate along with peptide calibration standards and BSA as a control and allowed to dry [18]. Peptide Mass Fingerprinting (PMF) was used for protein identification and the spectra were analysed in Flex Analysis and BioTools software from BrukerDaltonics. Peak lists were submitted to the MASCOT (http://www.matrixscience.com) search engine for analysis. Protein sequence database searching was performed on the NCBInr database. The general search parameters were: monoisotopic molecular masses, all species, variable modification was methionine oxidation and global modification was carbamidomethyl cysteine alkylation, with a mass tolerance of 100 ppm. 

### Identification of immunogenic membrane proteins from *B. cenocepacia* and *B. multivorans* by Bcc infected CF patient sera

The gels were manually aligned on the top of blots by using chaperonin GroEL protein as standard marker as it was clearly identified in all four strains. GroEL has molecular mass of 57 kDa and pI 5 was clearly detectable in blots probed with patient sera. A second protein, porin (38 kDa, pI 9.5) was clearly identified in Coomassie blue stained gels from all 4 strains. Both of these proteins acted as a landmark proteins in the analysis. The identified proteins had high sequence coverage in mass spectrometric analysis (i.e. greater than 50% in the majority of cases and the highest is 90%). 

## Results

### Comparison of proteins from the two *B. cenocepacia* strains, BC-7 and C1394

**Figure 1:**
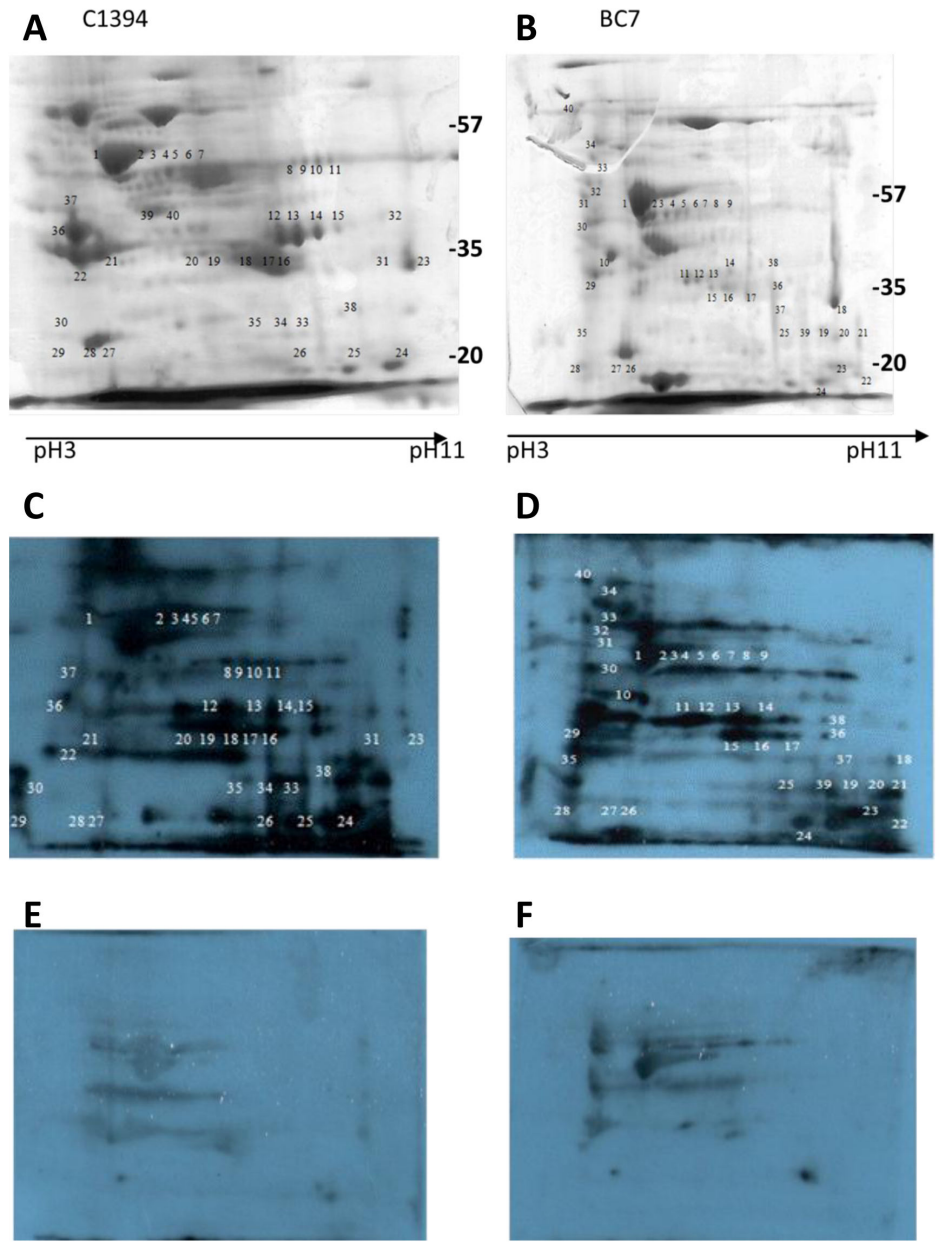
Representative Coomassie blue stained 2-D gel (pI 3-11) of *B. cenocepacia* strains, C1394 (A) and BC-7 (B) and the corresponding Western blots probed with pooled patient serum from Bcc colonised patients (C and D) or with serum from patients with no history of Bcc colonisation (E and F). Each gel was prepared with 120 µg membrane protein preparations extracted from 18 h cultures grown at 37 °C, focused on IEF strips (pH 3 to pH 11) separated on 12% SDS-PAGE gels, , blotted and probed the respective sera and detected with anti-human IgG. The corresponding spots on the gel were excised and identified by MALDI-ToF/MS analysis. The numbers represent the proteins spots referred to in the text. The approximate positions of molecular mass markers (kDa) are indicated.

The two *B. cenocepacia* strains were selected as examples of a piliated strain (BC-7) and non-piliated strain (C1394). About 100 protein spots were separated on each gel (Figure 1), among these 60 kDa chaperonin GroEL (spot 1 Figure 1A and 1B) and 38 kDa Porin (spot 23, Figures 1A and 18 in Figure 1B) showed a consistent pattern on both gels. Comparison of the Coomassie blue images indicates that there were considerable differences between the proteins isolated from each of these two *B. cenocepacia* strains. In particular, thiolase isozymes (Spots 13 & 14 Figure 1A) were clearly identified in C1394 but were not readily detectable on the BC-7 gels. The enzymes like inosine 5'-monophosphate dehydrogenase (IMPDH) (Figure 1A, spot 8, 9, 10 and 11) and alcohol dehydrogenase (spots 16,17 and 18) were also strongly expressed in C1394 cells and were absent in BC-7. In addition, outer membrane protein OprM (spots 2, 4, 5 Figure 1B) and outer membrane protein A precursor (spot 23, Figure 1B) were expressed in BC-7 cells, but were not visible on the C1394 gel (Figure 1A).

### Immunoblot analysis of *B. cenocepacia* membrane proteins using CF patient serum

When the proteins of the *B. cenocepacia* strains were probed with Bcc+ serum to identify the immunogenic proteins, an abundance of proteins was apparent (Figure 1C and D). Over forty different strongly immunogenic proteins were visible in blots from both strains, 32 of which were identified by MALDI-ToF MS (Table 1). The most immunoreactive proteins spanned a broad range of pI values between 4.5 and 10 and molecular mass from 20 to 60 kDa. There were approximately 12 proteins with a pI value between 7 to 11 in the two *B. cenocepacia* strains. 

**Table 1:** Immunogenic proteins observed in *B. cenocepacia* strains as identified by 2-D gels and MALDI-ToF mass spectrometry.

**Protein name**	**Functional category**	**Accession Number**	**Predicted Subcellular location^a^**	**Localisation score**	**MW(kDa) /p*I*^b^**	**Seq. coverage (%)**	**Score^c^**	**Strain**
alkyl hydroperoxide reductase/ Thiol specific antioxidant	Cellular processes	gi|107028881	CP	9.97	20.5/4.9	44-55	84-118	BC-7, C1394
chaperonin GroEL	Protein stabilisation	gi|161525697	CP	9.97	57.1/5.0	46-66	200-217	BC-7, C1394
dipeptide transporter ATP-binding subunit	Transport and binding	gi|107023972	CPM	7.88	37.5/9.5	26.3-42	63-107	BC-7, C1394
divergent AAA domain protein	Transcription	gi|323143202	CP	8.96	57.1/7	21-27	86-92	BC-7, C1394
DNA-directed RNA polymerase subunit alpha	Transcription	gi|161523455	CP	9.97	35.7/5.6	43-60	81-172	BC-7, C1394
elongation factor Tu	Transcription	gi|78064909 gi|221213513	CP	9.97	43.1/5.3	27-61	52-113	BC-7, C1394
phosphopyruvate hydratase	Energy metabolism - glycolysis	gi|134296288	CP	9.97	45.9/4.6	46-50	139-119	BC-7, C1394
porin	Transport and binding protein	gi|107025986	OM	10.0	37.7/9.7	40-65	119-139	BC-7, C1394
putative ubiquinone biosynthesis protein UbiB	Unknown function	gi|161523750	CPM	7.88	59.9/9.3	21.7-22	66	BC-7, C1394
30S ribosomal protein S1	Protein synthesis	gi|161525427	CP	9.97	62.2/4.9	22-26	88-110	BC-7
Enoyl CoA hydratase	Cell-cell communication	gi|311104936	CP	9.97	29.8/4.8	32-34	102-121	BC-7
EvpB family type VI secretion protein	Secretion protein	gi|161526098	CP	9.26	55.0/5.1	52-57	91-97	BC-7
F0F1 ATP synthase subunit alpha	Energy metabolism - electron motive force	gi|161523284	CP	9.97	55.8/5.5	32.7-62.1	96-185	BC-7
Hypothetical protein BCAS0292	Unknown function	gi|197295141	U	U	19.9/5.6	56-84	180-208	BC7
LysM domain/M23 peptidase domain protein	Peptidoglycan binding	gi|221198090	OM	9.93	30.0/9.9	37-44	66-83	BC-7
OmpA/MotB domain-containing protein	OM protein	gi|115350969	OM	9.93	24.1/10.1	55-56	105-328	BC-7
outer membrane efflux protein OprA	Transport and binding	gi|221212959	OM	10.0	52.9/9.1	37-44	66-83	BC-7
outer membrane protein OprM	Transport and binding	gi|221200195	OM	10.0	54.3/5/7	36.9-43	112-524	BC-7
oxidoreductase, aldo/keto reductase family	Energy metabolism	gi|221201924	CP	9.97	38.2/5.8	44-56.8	95-114	BC-7
Peptidoglycan-associated lipoprotein	Transport	gi|357936457	OM	10.0	18.5/6.6	23-30	58	BC-7
Polyribonucleotide nucleotidyltransferase	RNA binding and catabolic processing	gi|221211731	CP	9.97	77/5.2	24.9-28	94-111	BC-7
Putative ferritin DPS-family Binding protein	DNA binding	gi|206561636	CP	9.26	18.0/5.7	46-56	100-124	BC-7
putative outer membrane porin	Transport and binding protein	gi|221196031	OM	10.0	38.0/9.5	39.0-49.9	109-685	BC-7
ubiquinone/menaquinone biosynthesis methyltransferase	Metabolic processes	gi|107023667	CP	9.97	27.1/9.1	30-54	81-130	BC-7
acetyl-CoA acetyltransferase	Metabolic processes	gi|107023705	CP	9.97	40.5/6.7	19-51	100-273	C1394
Zn-dependent alcohol dehydrogenase	Energy metabolism glycolysis, gluconeogenesis, chloroalkane metabolism	gi|254248364	CP	9.97	38.2/6.3	52-57	102-204	C1394
aldehyde dehydrogenase	Energy metabolism / glycolysis, gluconeogenesis, chloroalkane metabolism	gi|170737358	CP	9.97	54.2/5.8	20.5-34.2	71-102	C1394
cell shape determining protein, mreb/mrl family	Cell membrane	gi|319761104	CP	9.97	37.0/5.1	24	82-94	C1394
F0F1 ATP synthase subunit beta	Energy metabolism - ATP-proton motive force interconversion	gi|107024491	CP	9.12	50.7/5.1	34-40	82-104	C1394
HAD family hydrolase	Unknown function/ enzyme of unknown specificity	gi|107022869	CP	9.97	24.5/5.3	48.8-57	66-101	C1394
hypothetical protein Bcen_2492	Hypothetical protein	gi|107024037	CP	9.97	32.0/8.9	32-46	71-140	C1394
inosine 5'-monophosphate dehydrogenase	Unknown function/ enzyme of unknown specificity	gi|206560426 gi|115352075	CP	9.97	52.2/7.1	32.3-51.4	80-150	C1394
Thiolase	Unknown function/ enzyme of unknown specificity	gi|254248092	CP	9.97	40.5/7.8	37.9-57.1	98-196	C1394

^a^ Predicted subcellular localisation determined using PSORTb V3 (www.psort.org) [19]. OM: outer membrane; CPM: Cytoplasmic membrane; CP: cytoplasmic, U: unknown.

^b^ Theoretical molecular mass (kDa) and isoelectric point were determined by Mascot.

^c^ Ranges represent MS/MS ion scores determined by peptide mass fingerprinting [61]. Only scores which were deemed to be significant by Mascot (p<0.05) are reported.

Nine spots were identified as immunoreactive in both strains, including highly immunoreactive proteins such as DNA-directed RNA polymerase subunit alpha corresponding to spots 19 and 20 in C1394 (Figure 1C) and spots 11 and 12 in BC-7 (Figure 1D). Chaperonin GroEL (spot 1 in both blots) and phosphopyruvate hydratase (spot numbers 37 and 10 in C1394 and BC-7 blots, respectively) (Table 1) were also immunogenic in both strains. The 38 kDa Porin (spot 18 in BC-7; 23 in C1394), which was used as a landmark spot was weakly immunogenic in both *B. cenocepacia* strains. 

A considerable number of immunogenic proteins appeared to be unique across all three experiments to an individual *B. cenocepacia* strain, such as thiolase (spot 13), aldehyde dehydrogenase (spot 5), IMPDH (spot 8, 9, 10, 11), and Zn-dependent alcohol dehydrogenase were specific to the non-piliated *B. cenocepacia* C1394 strain. In contrast, outer membrane efflux protein OprA (spot 9) and ATP synthase F1 alpha subunit (spot 6) are examples of immunogenic proteins which were identified on BC-7 immunoblots but not C1394 immunoblots (Table 1). These immunogenic proteins were identified in duplicate samples taken from three independent experiments. Certain protein spots on the gels were weakly stained, in contrast to the clear and strong signals on immunoblot, with the result that several immunogenic proteins could not be identified. It is clear that these proteins generated an abundance of antibodies in CF patient serum despite being expressed at an apparently low level in the Bcc strains. The subcellular localisation of the immunogenic proteins was predicted using PSORTb V3 (http://www.psort.org)[19]. Of the 32 immunogenic proteins in Table 1, only 6 were predicted to be outer membrane proteins (OMPs), while 2 were predicted to be on the cytoplasmic membrane. The remaining proteins were predicted to be cytoplasmic or were predicted as “unknown”. 

In comparison to the blots probed with Bcc^+^ serum, there was a considerable difference in the number and level of immunoreactive proteins in blots probed with Bcc- serum (Figure 1E and 1F). Due to the high incidence of *P. aeruginosa* colonisation among adult CF patients, all sera used in the control blots were seropositive for *P. aeruginosa*. These showed a much weaker antibody response, indicating that the majority of immunogenic proteins in Bcc were not expressed by *P. aeruginosa* or were not homologous to *P. aeruginosa* proteins. Certain proteins were weakly cross-reactive, for example, chaperonin GroEL, probably due to homology between certain conserved *P. aeruginosa* antigens and Bcc proteins. The low signal intensities on the immunoblots probed with Bcc- serum confirm a high concentration of specific antibodies are produced in serum specifically in response to Bcc colonisation. 

**Figure 2:**
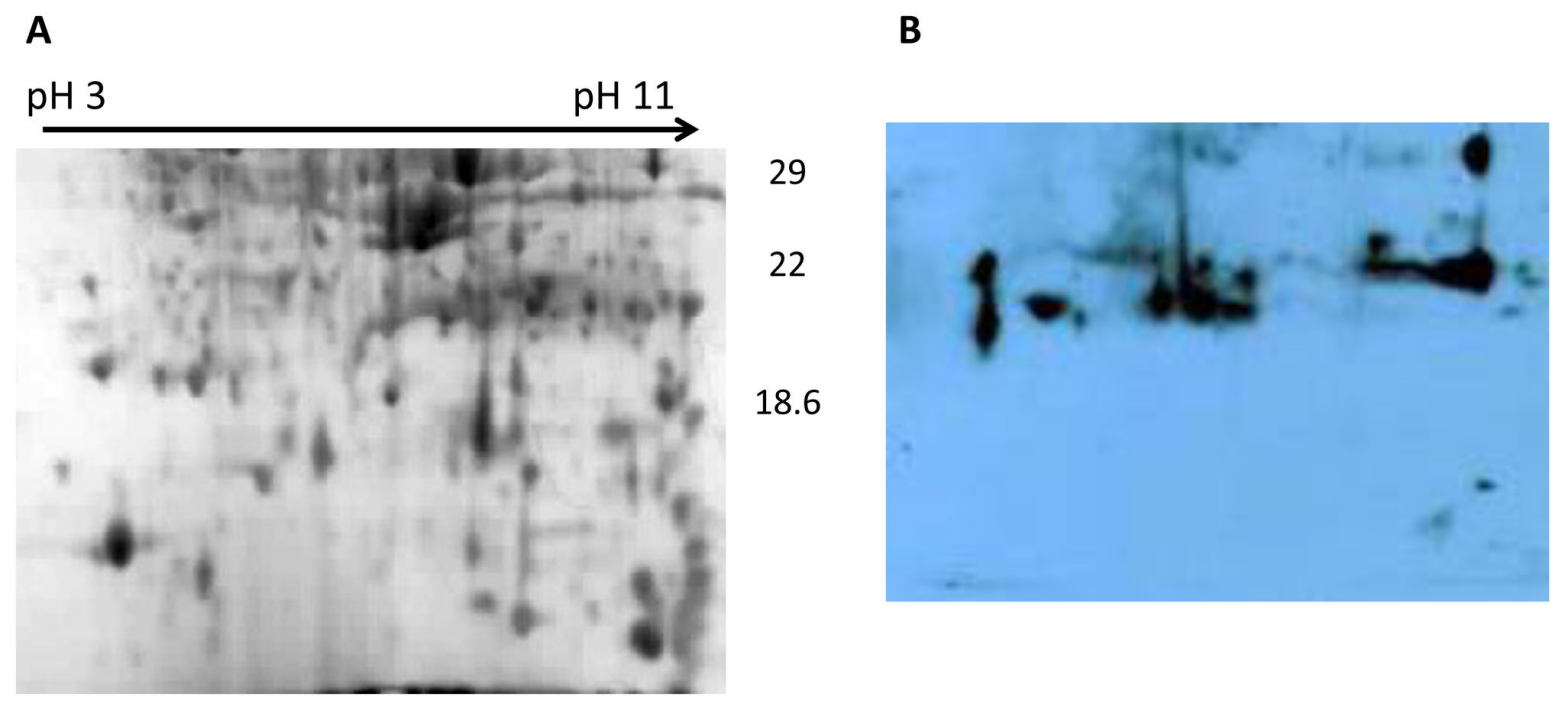
Representative Coomassie blue stained 15% 2-D gels and corresponding blots for *B. cenocepacia* strain BC-7. Each gel was prepared with 120 µg membrane protein preparations extracted from 18 h stationary phase cultures, focussed on pH gradient strips (pH 3 to pH 11), separated on 15% SDS PAGE gels and either stained with Coomassie blue (A) or probed with serum from Bcc colonised CF patients (B). The approximate positions of molecular mass markers (kDa) are indicated beside the Coomassie blue stained gel.

There were several immunogenic proteins present in both blots at the low MW region (below 25kDa). These were not well resolved on 12% gels but were identifiable using 15% gels (Figure 2). Additional immunogenic proteins were identified on these gels and blots. These include Enoyl coA-hydratase, putative ferritin DPS family DNA binding protein, a hypothetical protein product of gene BCAS0292 and ubiquinone/ menaquinone biosynthesis methyltransferase.

### Comparison of protein expression in the two *B. multivorans* strains

**Figure 3:**
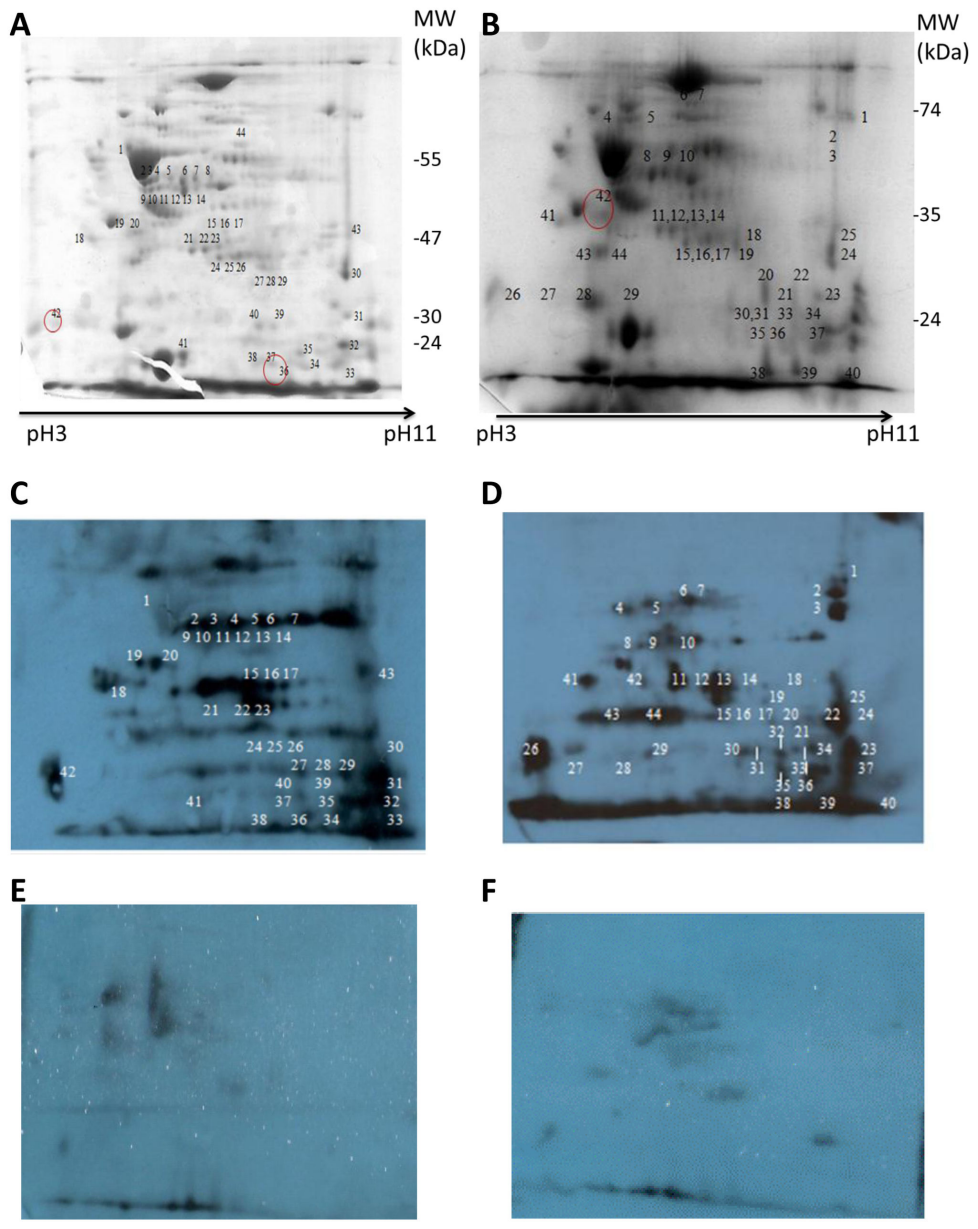
Representative Coomassie blue stained 2-D gel (pI 3-11) of *B. multivorans* strains, LMG13010 (A, E) and C1962 (B) and the corresponding Western blots probed with pooled patient serum from Bcc colonised patients (C and D) or with serum from patients with no history of Bcc colonisation (E and F). Each 12 % 2-D gel was prepared with membrane protein preparations extracted from 18 h cultures grown at 37° , and focused on IEF strips (pH 3 to pH 11), blotted and probed the respective sera and detected with anti-human IgG. The corresponding spots on the gel was excised and identified by MALDI-ToF/MS analysis. The numbers represent the proteins spots referred to in the text. The images shown are representative of three individual experiments.

Approximately 100 distinguishable protein spots were identified on the Coomassie blue stained 2-D gel of both C1962 and LMG13010 (Figure 3). There was more similarity between the proteins of the two *B. multivorans* strains than there was between the two *B. cenocepacia* strains. However, there were certain proteins that were present in the LMG13010 gel and absent in the C1962 gel and *vice versa*. For example, transketolase and aminotransferase respectively, were strongly expressed in C1962 gels but were not detectable in LMG13010 gels. The migration pattern of chaperonin GroEL (spot 6 and 7 in Figure 3A and spot 45 in Figure 3B) and 38kDa porin (spot 47 in Figure 3A and spot 24 and 25 in Figure 3B) were comparable on all *B. multivorans* gels, consistent with the observations in *B. cenocepacia* and again these were used to align the gels and blots. Further similarity across the two species was indicated by elongation factor Tu, acetyl-CoA acetyltransferase and cell shape determining protein being identified in *B. cenocepacia* and in *B. multivorans*.

### Immunoblot analysis of *B. multivorans* membrane proteins using CF patient serum

**Figure 4:**
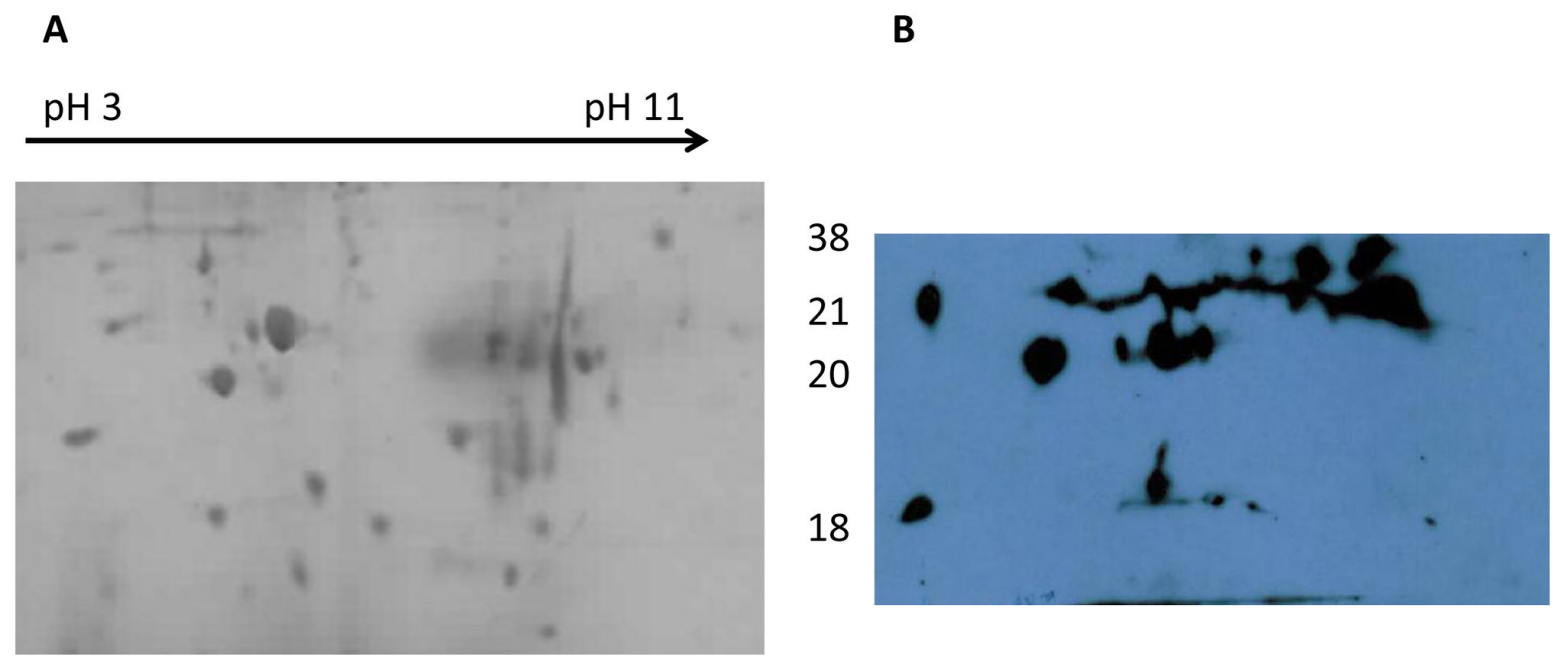
Representative Coomassie blue stained 15% gels and corresponding blots for *B. multivorans* strain LMG13010. Each gel was prepared with 120 µg membrane protein preparations extracted from 18 h stationary phase cultures, focussed on pH gradient strips (pH 3 to pH 11), separated on 15% SDS PAGE gels and either stained with Coomassie blue (A) or probed with serum from Bcc colonised CF patients (B). The approximate positions of molecular mass markers (kDa) are indicated beside the Coomassie blue stained gel.

Immunoblotting of *B. multivorans* proteins also showed a clear difference in pattern and intensity of immunogenic proteins blots probed with Bcc+ serum (Figure 3C, 3D) relative to those probed with Bcc- serum (Figure 3 E and 3F). Among the 100 proteins identified on the Coomassie blue stained gels, 33 proteins were detected by CF patient serum antibodies and showed strong signals on the immunoblot (Table 2). Seventeen immunoreactive proteins were identified in both strains, including proteins such as LysM domain/M23 peptidase, DNA directed RNA polymerase and F_0_F_1_ ATP synthase. There were five immunogenic proteins which were clearly observed on LMG13010 blots (of 12% gels) and not observed in C1962 blots. In addition, three proteins that were reproducibly identified on the C1962 blots were not identified on LMG 13010 blots. Further analysis of low molecular mass proteins on 15% gels (Figure 4) allowed the identification of three additional immunoreactive proteins, GTP cyclohydrolase, Hcp1 secretion effector, hydroperoxide reductase and type VI secretion protein (Table 2). As with *B. cenocepacia*, due to low abundance of certain proteins on the gels, some immunoblot spots could not be identified unambiguously in the 2-D gel of both strains. 

**Table 2:** Immunogenic proteins observed in *B. multivorans* strains as identified by 2-D gels and MALDI-Tof mass spectrometry.

**Protein name**	**Functional category**	**Accession Number**	**Subcellular location** ^a^	**Localisation score**	**MW(kDa)/p*I*^b^**	**Seq. coverage (%)**	**Score^c^**	**Strain**
30S ribosomal protein S1	Protein synthesis	gi|161525427	CP	9.97	62.2/4.9	32.1-39	132-160	LMG13010, C1962
cell shape determining protein, mreb/mrl family	Cell envelope	gi|319761104	CP	9.97	37.0/5.7	26-34	92-128	LMG13010, C1962
chaperonin GroEL	Protein stabilisation	gi|161525697	CP	9.97	57.1/5	24-65.5	111-310	LMG13010, C1962
DNA-directed RNA polymerase subunit alpha	Transcription	gi|161523455	CP	9.97	35.8/ 5.6	41.8 -63.4	167-235	LMG13010, C1962
elongation factor Tu	Transcription	gi|221213513	CP	9.97	41.8/5	37.9-49	96-122	LMG13010, C1962
F0F1 ATP synthase subunit alpha	Energy metabolism - electron motive force	gi|161523284	CP	9.97	55.8/5.5	32.7-62.1	96-185	LMG13010, C1962
Hypothetical protein 0835	Hypothetical protein	gi|161524014	P	9.84	25.6/9.6	80-89	28.5-68.1	LMG13010, C1962
isocitrate lyase	Energy metabolism - Glyoxylate cycle	gi|161524402	CP	9.97	47.9/5.7	27.1-51.4	71-133	LMG13010, C1962
LysM domain/M23 peptidase domain protein	Peptidoglycan binding protein	gi|221198090	OM	9.93	30/9.9	40.7-46	71-124	LMG13010, C1962
nitrate reductase, beta subunit	Energy metabolism / anaerobic	gi|221198557	CPM	9.82	59/5.8	26.8-36	79-127	LMG13010, C1962
OmpA/MotB domain-containing protein	Outer membrane protein	gi|115350969	OM	9.93	24.1/10.1	34-49.1	84-408	LMG13010, C1962
outer membrane protein OprM	Transport and binding	gi|221200195	OM	10.0	54.3/5.7	26.9-41.4	121-245	LMG13010, C1962
oxidoreductase, aldo/keto reductase family	Energy metabolism	gi|221201924	CP	9.97	38.2/5.8	52.0-78.7	114-225	LMG13010, C1962
porin	Transport and binding	gi|161520486	OM	9.95	38.1/9.5	45-68.6	124-241	LMG13010, C1962
putative outer membrane porin	Transport and binding	gi|221196031	OM	10.0	38./9.5	42-55.4	105-434	LMG13010, C1962
soluble lytic murein transglycosylase	Cellular processes	gi|294634704	U	U	72.6/9.9	25-26	94-97	LMG13010, C1962
type II citrate synthase	Energy metabolism	gi|161521186	CP	9.97	48.9/6.3	30.7-34.5	77-129	LMG13010, C1962
dipeptide transporter ATP-binding subunit	Transport and binding	gi|107023972	CPM	7.88	37.5/9.5	36-44	84-98	LMG 13010
EvpB family type VI secretion protein	Secretion system	gi|161526098	CP	9.26	55/5.1	41.7-68	118-198	LMG 13010
GTP cyclohydrolase I	Haemolysis	gi|161521826	CP	9.97	23.5/9.0	35-67	81-147	LMG13010
Hcp type VI secretion system effector	Secretion system	gi|161526097	EC	10.0	18.5/6.9	77-82	99-154	LMG13010
Heat shock protein Hsp20	Protein stabilisation	gi|161525226	CP	8.96	15.1/4.9	58-89	96-205	LMG13010
Hydroperoxide reductase	Cellular processes	gi|53719707	CP	9.97	20.4/4.9	44-54	92-154	LMG13010
Outer membrane lipoprotein precursor	Transport	gi|30314414	OM	10.0	16.5/5.7	50	87	LMG13010
Peptidoglycan binding LysM	Peptidoglycan binding	gi|221198154	U	U	16.3/4.6	62-71	98-116	LMG13010
phosphonate-transporting ATPase	Transport binding protein	gi|319955500	CPM	7.88	27.3/5.6	33-43	85-87	LMG 13010
phosphopyruvate hydratase	Energy metabolism - glycolysis	gi|161524338	CP	9.97	45.9/4.6	28.3-60	125-168	LMG 13010
recombinase A	Gene expression	gi|161523830	CP	9.97	38.3/4/9	30.5-36.4	66-73	LMG 13010
succinate dehydrogenase flavoprotein subunit	Energy metabolism	gi|161521189	CPM	7.88	65/6.2	31.-37.1	123-146	LMG13010
Type VI secretion protein	Secretion system	gi|161526099	CP	9.97	19.2/5.1	46-61	110-141	LMG13010
Acetyl CoA Acetyltransferase	Cell metabolism	gi|221198056	CP	9.97	40.7/7.8	40.2-49.9	78-109	C1962
Dihydrolipoyl dehydrogenase	Fatty acid synthesis	gi|221214787	CP	9.97	51.9/5.3	31.9-39	98-103	C1962
Dyp-type peroxidase family	Unknown function/ enzyme of unknown specificity	gi|221207824	CP	8.96	37.2/4.8	23-61	114-246	C1962

^a^ Predicted subcellular localisation determined using PSORTb V3 (www.psort.org) [19]. OM: outer membrane; CPM: Cytoplasmic membrane; CP: cytoplasmic; P: Periplasmic; U: unknown

^b^ Theoretical molecular mass (kDa) and isoelectric point were determined by Mascot.

^c^ Ranges represent MS/MS ion scores determined by peptide mass fingerprinting [61]. Only scores which were deemed to be significant by Mascot (p<0.05) are reported.

### Immunogenic proteins common to both species

**Figure 5:**
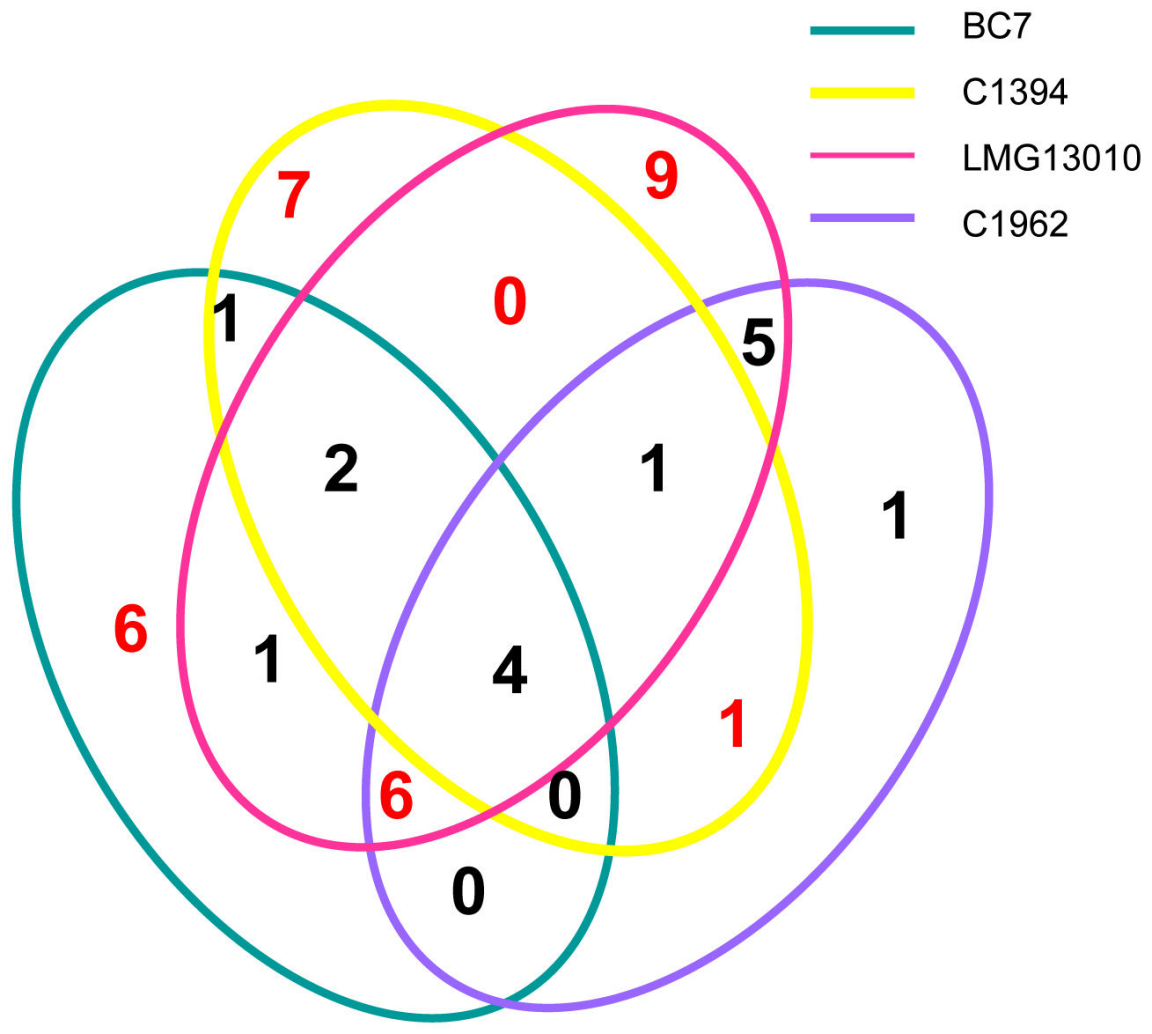
Venn diagram illustrating the numbers of immunogenic proteins identified which were common among strains examined from each species analysed.

DNA-directed RNA polymerase, GroEL, elongation factor Tu and the 38 kDa porin were common to all four strains (Figure 5). Furthermore, there were a total of 15 immunoreactive proteins which were common across both species and the similarities have been compared in Table 3. In the case of seven proteins, the accession number identified on MALDI was the same one for both species, for example, for chaperonin GroEL, the corresponding *B. multivorans* protein had the highest score for all 4 strains by MASCOT. Six of the other 15 proteins had sequence identities which were 97% or greater across the two species. The least similar protein among this fourteen was porin; BLAST analysis revealed the identity was only 79% [20]. The close similarity of these cross-reactive antigens highlights the potential for these antigens as protective antigens for both species. 

**Table 3:** Immunogenic proteins shared between *B. cenocepacia* and *B. multivorans* and their sequence similarity.

**Protein name**	**Accession Number(s)**	**Subcellular location^a^**	**Localisation score**	**Identities (%)**[62]
chaperonin GroEL	gi|161525697*	CP	9.97	100%*
DNA-directed RNA polymerase subunit alpha	gi|161523455*	CP	9.97	100%*
elongation factor Tu	gi|78064909 gi|221213513	CP	9.97	371/384 (97%)
F0F1 ATP synthase subunit alpha	gi|161523284*	CP	9.97	100%*
LysM domain/M23 peptidase domain protein	gi|221212760 gi|221198090	OM	9.93	287/289 (99%)
OmpA/MotB domain-containing protein	gi|161525435 gi|115350969	OM	9.93	221/222 (99%)
Outer membrane lipoprotein precursor Peptidoglycan-associated lipoprotein	gi|30314414 gi|357936457	OM	10	125/149 (84%)
outer membrane protein OprM	gi|221200195*	OM	10.0	100%*
oxidoreductase, aldo/keto reductase family	gi|221201924*	CP	9.97	100%*
porin	gi|107025986 gi|161520486 gi|221196031	OM	10.0	292/368 (79%) (99%)
dipeptide transporter ATP-binding subunit	gi|107023972*	CPM	7.88	100%*
EvpB family type VI secretion protein	gi|161526098*	CP	9.26	100%*
Alkyl hydroperoxide reductase/ thiol specific reductase Hydroperoxide reductase	gi|107028881 gi|53719707	CP	9.97	179/182(98%)
phosphopyruvate hydratase	gi|134296288 gi|161524338	CP	9.97	423/427 (99%)
Acetyl CoA Acetyltransferase	gi|107023705 gi|221198056	CP	9.97	381/392(97%)

^a^ Predicted subcellular localisation determined using PSORTb V3 (www.psort.org)[19] OM: outer membrane; CPM: Cytoplasmic membrane; CP: cytoplasmic; P: Periplasmic; U: unknown

* Accession number identified was identical for all strains, therefore, 100% identity inevitable.

## Discussion

This study identified a wide range of proteins expressed by the two most common *Burkholderia* species that colonise CF patients during infection. Although an analysis of the immunogenic proteins in the secretome of another member of the Bcc, *B. cepacia*, has been reported [21], this species represents only 2% of Bcc infection among CF patients [22]. Furthermore that study utilised mouse serum, rather than CF patient serum. There has been no study to examine or identify the membrane proteins expressed by any Bcc species during human infection. In order to address this gap in our knowledge we examined the immunoreactive proteins expressed in membrane protein preparations from the two most clinically relevant species of the Bcc, comparing four strains. Previous immunoproteomic studies on *P. aeruginosa* and *N. meningitidis* reported that there were striking differences in the antigens recognised by sera from different patients [12,13]. We wanted to get a global view of immunogenic proteins in both clinically relevant species of Bcc and used pooled serum from Bcc-colonised patients to avoid patient-specific effects. Stationary phase cultures were used as this growth phase best resembles the physiological state of bacteria which chronically colonise the lung. Studies carried out using alternative culture conditions that associated are also associated with the CF lung, including low pH and hypoxia could well highlight additional immunogenic proteins. 

 This proteomic analysis has reproducibly identified 44 immunogenic proteins across both species. Among these proteins, while 14 proteins (Table 3) were identified in both *B. multivorans* and *B. cenocepacia*, the number of proteins identified as immunogenic in all four strains examined was surprisingly small: GroEL, 38kDa porin, DNA-directed RNA polymerase (RNAP) and EF-Tu A total of 14 proteins were exclusive to *B. cenocepacia* only, while 15 were identified in *B. multivorans* only. Given inter-patient differences observed in other immunoproteomic studies, all seven patients are unlikely to produce antibodies against all four conserved proteins and therefore more biological variability is likely. Considerable differences in immunoreactive proteins were identified in the two *B. cenocepacia* strains. These two strains, BC-7 and C1394 represent different lineages within the *B. cenocepacia* species, as determined by *recA* gene sequence and referred to as IIIA and IIIB, respectively. This strongly suggests that a multi-component vaccine would be needed to protect CF patients from both *B. multivorans* and *B. cenocepacia*. 

Vaccination studies against CF pathogens have predominantly focussed on *P. aeruginosa* as it is the leading cause of respiratory infections in the CF population. There have been only two *in vivo* mouse vaccination studies reported against Bcc, both of which involved unpurified membrane proteins preparations. Bertot et al. described the protection of mice against either *B. cenocepacia* or *B. multivorans* challenge following nasal immunisation with an enriched membrane proteins preparation administered with a mucosal adjuvant [23]. More recently mice were protected from *B. cenocepacia* using enriched membrane proteins administered intranasally as a nanoemulsion preparation [24]. Both studies demonstrate the potential for membrane proteins as protective antigens, and although the 17 kDa membrane protein was identified as an immunodominant antigen in the latter study, identification of any other antigens that elicited these protective responses and may represent protective antigens for in a multi-subunit vaccine formulation have not been elucidated to date [24]. 

 RNAP was identified as immunoreactive in all four strains examined. It has previously been shown to be immunogenic in *B. pertussis* (BP642) and *Chlamydia trachomatis* [25]. The RNAP alpha subunit (BCAL0260) was upregulated in *B. cenocepacia* in response to low pH in recent studies [26]. It was differentially altered in *B. pertussis* during iron starvation and was suggested to be associated with virulence in that organism [27]. Many virulence regulators have been shown to interact directly with RNAP, including those in *F. tularensis* [28] and *Vibrio vulnificus* [29]. RNAP may also contribute to the activation of invasion genes in *Salmonella enterica* [30]. We and others have shown that Bcc can invade human lung epithelial cells [31,32] and immunogenicity of RNAP in CF patients indicates that it could also play an active role in the pathogenicity of Bcc, and may be involved in its intracellular invasion. 

Elongation factor Tu (EF-Tu) is the another immunogenic protein which was common to all four strains. It was previously identified as an immunoreactive protein in the secretome of another Bcc species, *B. cepacia* [21]. Although membrane proteins preparations were used in this study, it is clear that many of the immunogenic proteins identified were predicted to be cytosolic in cellular localisation. Riedel et al. first reported a rigorous analysis of the proteome of a *B. cenocepacia* strain and highlighted the overlap of proteins from distinct subcellular locations [33]. Of the 304 intracellular proteins that they identified, 46 were also located in either the extracellular or surface fractions. EF-Tu was reported as being highly abundant in *B. cenocepacia* and was present in all three fractions examined (intra-, extra- cellular and surface) [33]. The strong humoral responses of intracellular proteins among Bcc colonised CF patients in our study, indicates that the host immune system is exposed to these proteins either due to cell death or secretion via membrane bound vesicles. It is likely that the latter case is involved in the host recognition of EF-Tu, since it was secreted in outer membrane vesicles of *Burkholderia pseudomallei* [14]. It was also shown to reduce lung bacterial loads following challenge with a related organism *B. thailandensis* when used to immunise mice [14]. As with RNAP, EF-Tu was also shown to be upregulated in *B. cenocepacia* in response to low pH, but not low oxygen or low iron, or nutrient deficient medium [26]. EF-Tu was also immunoreactive in *F. tularensis*, *Sh. flexineri*, *Bacillus anthracis* and *P. aeruginosa* and binds to host proteins, such as fibrinogen and plasminogen [34-37]. Given that it was identified in both *B. multivorans* and *B. cenocepacia*, the role of EF-Tu in Bcc virulence warrants further investigation. 

Chaperonin GroEL was also immunoreactive across all four strains examined. It has been identified as immunogenic in many species including *Shigella flexineri*, *Yersinia enterocolitica, B. pseudomallei* and *P. aeruginosa* [12,34,38,39]. This stress response protein has been shown to be upregulated in leptospires cultured following *in vivo* infection relative to those cultured in vitro [40]. It has been associated with intracellular invasion of *Legionella* [41]. This well conserved protein was protective against lethal *S. pneumoniae* challenge [42] and conferred 100% protection against *Bacillus anthracis* infection [43] in mice. Although GroEL is abundant in all four Bcc strains examined, given its level of expression, it is less immunogenic than many other immunoreactive proteins identified and may not be relevant as a virulence factor nor be a good vaccine candidate for this species. 

Although 38 kDa porin (pI 9.5-9.7) served as a landmark protein, it was also weakly immunogenic across all four strains and consequently is not likely to represent either a major virulence protein or a vaccine candidate in Bcc. Furthermore, it was the protein with the lowest sequence similarity between *B. cenocepacia* and *B. multivorans*, relative to the other 13 common proteins. Another porin, OprM was considerably immunogenic, but was only identified in three strains. This 54.3 kDa porin is a component of the MexA-MexB-OprM efﬂux system, which was found to mediate multidrug resistance in *Ps. aeruginosa* [44]. 

A number of other immunoreactive Bcc proteins have been previously associated with binding of other pathogens to host proteins, including phosphopyruvate hydratase (Pph), which was identified in three of the four strains. Pph, also known as enolase, was immunogenic in other invasive organisms, such as *S. pneumoniae* [9,10], *Campylobacter concisus* [45] and *Borrelia burgdorferi* [46]. It has been shown to bind to plasminogen and plays a role in the pathogenesis of a variety of organisms, including *Bo. burgdoferi* [46], oral streptococci [47] and *Neisseria meningitides* [13]. Enolase expressed by *Bo. burgdoferi* lacks a signal peptidase cleavage site and cell wall anchor sequences, but was associated with outer-membrane vesicles which may be related to plasmin fixing to the peribacterial environment [46]. Another host binding protein, lysM domain/M23 peptidase domain protein was identified in all strains except C1394. A protein with these domains has been previously identified as a fibronectin-binding protein involved in *T. denticola*-associated with periodontal disease [48]. A LysM domain protein was also identified among proteins secreted from *S. pneumoniae* [9]. Further analysis of host binding proteins such as these may give better insights in to the pathogenesis of Bcc. 

The 38 kDa Oxidoreductase-aldo/ketoreductase (38 kDa, pI 5.8) was identified among the immunogens in both species. Although this enzyme has not been identified in previous immunoproteomics studies of other bacterial pathogens, a member of the aldo/ ketoreductase family (33 kDa) from *Trypanosoma cruzi* has been identified as a major immunogen in alkaline fractions of *T. cruzi*, generating potent B-cell responses in mice [49]. Another aldo/ketoreductase has been associated with uropathogenic *E. coli* [50]. F_0_F_1_ ATP synthase subunits were immunoreactive in all four strains. The alpha subunit (55 kDa) of all strains except C1394 was identified as immunoreactive, while the beta subunit (50kDa) was identified as immunogenic in C1394. Both subunits were listed as immunoreactive proteins in a dominant serotype of *S. flexineri* [34]. The alpha subunit was one of three proteins found to be immunoreactive across all sera from 10 patients with Crohn’s disease when used to probe *C. concisus* blots [45]. In addition, the alpha subunit of ATP synthase may also be involved in the endothelial invasion mechanism of *Bartonella hensellae* [51]. The F_0_F_1_ ATP synthase beta subunit was identified among 18 immunogenic proteins in *Brucella abortus* [52] and in two *Bordetella pertussis* vaccine strains [53,54]. ATP synthase beta subunit of the periodontitis-associated pathogen *Aggregatibacter acinomycetemcomitans* has been shown to bind to human interleukin-1β and may be associated with the suppression of local inflammation [55]. The finding that two subunits of this conserved protein are immunoreactive in Bcc warrants further investigation as to its role in pathogenesis. Another immunoreactive protein identified in both *B. cenocepacia* strains was Alkyl hydroperoxide reductase (AhpC). It shows 98% identity with another immunoreactive protein identified in *B. multivorans*, hydroperoxide reductase (Blast-P) [20]. AhpC was down regulated in a persistent isolate of *B. cenocepacia* C1394 during murine infection and loss of this protein was associated with enhanced susceptibility to oxidative stress [56]. More recently Zlosnik et al. showed that AhpC was strongly overexpressed in a non-mucoid *B. cenocepacia* isolate during chronic infection and was suggested to play a role in resistance towards killing by phagocytes [57]. Furthermore, AhpC was an immunoreactive surface protein in another member of genus *Burkholderia*, *B. thailandensis* [14]. Another protein that was identified in both species as immunogenic in three of the strains was the dipeptide transporter ATP-binding subunit. This has also been identified as immunoreactive in *S. pneumoniae* and also identified by expression library immunisation in *B. mallei*, another related *Burkholderia* respiratory pathogen associated with glanders in horses [9,58]. 

Metabolic enzymes are often identified as immunoreactive in bacterial pathogens and may indicate a role in pathogenesis [15]. Citrate synthase was identified as immunogenic in both *B. multivorans* strains. Mutations in a putative citrate synthase gene in *B. cenocepacia* (BCAS207) resulted in reductions in biofilm formation and partial deficient virulence [59]. Furthermore, inosine monophosphate dehydrogenase (IMPDH) was reproducibly identified in *B. cenocepacia* strain C1394 only. IMPDH plays a role in virulence in the swine pathogen, *Streptococcus suis*, type 2; deletion mutants of this gene cannot form hyaluronic acid capsule and have a reduced rate of invasion [60]. Its potential role in virulence of *B. cenocepacia* remains to be elucidated.

Overall, several immunoreactive proteins have been identified in this study, which were previously identified as virulence proteins in other pathogens. Although many of these were common to both clinically relevant species, the number common to all four strains examined was minimal, highlighting the diversity across Bcc strains, which is a hallmark of Bcc. These immunoreactive proteins which were also shown to be involved in pathogenesis of other organisms warrant further investigation in order to elucidate the mechanisms of pathogenesis of this highly virulent opportunistic pathogen. The potential for these immunogenic proteins to be vaccine antigens remains unexplored to date. Antigens which were common to both *B. multivorans* and *B. cenocepacia* in addition to other Bcc species would be advantageous in terms of their potential to protect people with CF from a range of species with the Bcc. Furthermore, prior to development of any vaccine antigen any potential cross reactivity with commensal bacteria would need to be ruled out. Given the fact that Bcc has an intracellular component to its lifestyle, in addition to extracellular adhesion, the cellular responses of any antigens would need to be explored in addition to the humoral response in developing protective vaccines against this organism. 
